# FDK-Type Algorithms with No Backprojection Weight for Circular and Helical Scan CT

**DOI:** 10.1155/2012/969432

**Published:** 2012-02-16

**Authors:** A. V. Narasimhadhan, Kasi Rajgopal

**Affiliations:** Department of Electrical Engineering, Indian Institute of Science, Bangalore 560 012, India

## Abstract

We develop two Feldkamp-type reconstruction algorithms with no backprojection weight for circular and helical trajectory with planar detector geometry. Advances in solid-state electronic detector technologies lend importance to CT systems with the equispaced linear array, the planar (flat panel) detectors, and the corresponding algorithms. We derive two exact Hilbert filtered backprojection (FBP) reconstruction algorithms with no backprojection weight for 2D fan-beam equispace linear array detector geometry (complement of the equi-angular curved array detector). Based on these algorithms, the Feldkamp-type algorithms with no backprojection weight for 3D reconstruction are developed using the standard heuristic extension of the divergent beam FBP algorithm. The simulation results show that the axial intensity drop in the reconstructed image using the FDK algorithms with no backprojection weight with circular trajectory is similar to that obtained by using Hu's and T-FDK, algorithms. Further, we present efficient algorithms to reduce the axial intensity drop encountered in the standard FDK reconstructions in circular cone-beam CT. The proposed algorithms consist of mainly two steps: reconstruction of the object using FDK algorithm with no backprojection weight and estimation of the missing term. The efficient algorithms are compared with the FDK algorithm, Hu's algorithm, T-FDK, and Zhu et al.'s algorithm in terms of axial intensity drop and noise. Simulation shows that the efficient algorithms give similar performance in axial intensity drop as that of Zhu et al.'s algorithm while one of the efficient algorithms outperforms Zhu et al.'s algorithm in terms of computational complexity.

## 1. Introduction

In divergent beam ramp FBP reconstruction algorithms, it is known that the properties such as noise, resolution and divergent beam artifacts of reconstructed image are influenced by the position-dependent weight in the backprojection. The backprojection weight (which is related inversely to the distance of the source to the point being reconstructed) causes spatially nonuniform distribution of noise and resolution, and the divergent beam artifacts are more pronounced away from the rotation axis [1–3]. Many algorithms using different approaches have been proposed to address this issue. Pan [2, 4] used shift variant ramp filtering approach while Wang et al. [5] proposed spatially variant weighting in the backprojection. These approaches are computationally expensive. Recently, divergent beam FBP reconstruction algorithms have been proposed with inverse distance weight [6] and no backprojection weight [7, 8]. The reconstructed images with these algorithms have better properties in terms of reduced noise level and uniformity in noise distribution and uniformity in resolution as compared to the classical ramp FBP reconstruction algorithm. The fan-beam reconstruction algorithms with inverse distance backprojection weight have been extended to obtain 3D cone-beam (CB) FDK-type approximate reconstruction algorithms with improved noise and resolution properties [9]. On the other hand, algorithms have been developed to improve the noise performance with the modified redundancy weight in the projection domain for circular and helical CB CT [10–12]. The fan-beam reconstruction algorithms with no backprojection weight can be extended to obtain 3D CB FDK-type approximate reconstruction algorithms to improve noise and resolution properties. 

The most widely used practical method for CB reconstruction has been the approximate algorithm of Feldkamp, Davis and Kress (FDK) [13] originally proposed for circular scan CB reconstruction, and FDK has been extended to helical scan CB geometry by Wang et al. [14]. The circular scan trajectory geometry does not satisfy Tuy's data sufficiency condition on the projections for exact and stable reconstruction [15, 16] permitting only approximate reconstruction and also leads to intensity drop in the reconstructed images. Many methods have been suggested to give improvements in the image quality when reconstructing from circular CB data. Some of them use the iteration schemes to reconstruct the image with circular scan FDK as an intermediate result [17, 18]. These algorithms are computationally inefficient due to loss of FBP structure. Another approach to reduce the axial intensity drop is given by Hu [19] which adds, to FDK reconstruction, a correction term obtained from unused circular scan CB data. Zhu et al. [20] propose to further add a “missing term” to obtain a more accurate FDK reconstruction. The missing term is obtained by estimating the missing data, required by the data sufficiency condition, from the CB scan projection data. 

In this paper, two exact Hilbert FBP reconstruction algorithms with no backprojection weight for 2D fan-beam with equispace linear array detector geometry (complement of the equi-angular curved array detector) have been derived. Using these fan-beam Hilbert FBP inversion algorithms, Feldkamp-type reconstruction algorithms with no backprojection weight for 3D reconstruction for circular and helical scan trajectories are developed. Further, we present efficient algorithms to reduce the axial intensity drop for circular CB CT using the proposed FDK reconstruction algorithms with no backprojection weight. Simulation studies demonstrate the properties of the proposed reconstruction algorithms with no backprojection weight and the efficient algorithms for reducing axial intensity drop in 3D CB CT. We compare the performance of these algorithms with other algorithms in the literature.

## 2. Fan-Beam Algorithms with No Backprojection Weight for Equispace Linear Detector

In this section, we revisit the relation between the parallel beam projections and the fan-beam projections through the Hilbert kernel. Using this relation, we derive the fan-beam reconstruction formulae with no backprojection weight for equispace linear array detector, which are counterpart of the algorithms for the curved array detector [7, 8].

### 2.1. Parallel Beam Projections

**Figure 1:**
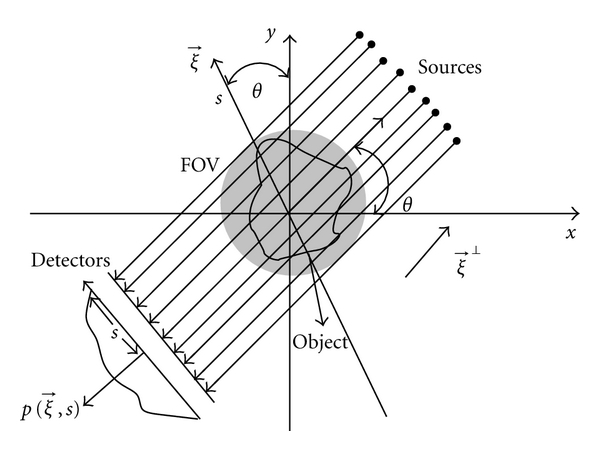
Parallel-beam geometry.

Let $f(\vec{x})$ denote the 2D object density function to be reconstructed where $\vec{x}={\left(x,y\right)}^{T}$. The parallel-beam projections of *f* are given by the expression

(1) $$\begin{array}{c} p\left(\vec{\xi },s\right)=\overset{}{\underset{{\mathbb{R}}^{2}}{\int }}f\left(\vec{x}\right)\delta \left(\vec{x}\cdot \vec{\xi }-s\right)d\vec{x} \end{array},$$

where *s* ∈ ℝ, $\vec{\xi }=(-\sin\theta ,\cos\theta )$ is any unit vector and *δ* is Dirac delta function. The delta function in (1) selects the value of $\vec{x}$ that satisfies $\vec{x}\cdot \vec{\xi }=s$, so that $p(\vec{\xi },s)$ is the integral of *f* on the line perpendicular to $\vec{\xi }$ at a distance *s* from the origin $\vec{x}=(0,0)$ as shown in Figure 1.

The inversion of *f* from its projection *p* using the FBP algorithm involves two steps. The first step is to filter the projection $p(\vec{\xi },s)$ by applying 1D ramp filter in *s*,

(2) $$\begin{array}{c} p_{R}\left(\vec{\xi },s\right)=\left(h_{R}*p\right)\left(\vec{\xi },s\right)=\overset{}{\underset{\mathbb{R}}{\int }}p\left(\vec{\xi },s^{\prime }\right)h_{R}\left(s-s^{\prime }\right)ds^{\prime }, \end{array}$$

where *h*_*R*_ = ∫_−*∞*_^*∞*^ | *σ* | exp⁡(*j*2*πσs*)*dσ* is the impulse response of |*σ*| filter. The ramp filter is seen as equivalent to the successive application of a derivative in *s* and Hilbert transform,

(3) $$p_{R}\left(\vec{\xi },s\right)=\left(h_{H}*\frac{\partial }{\partial \text{s}}p\right)\left(\vec{\xi },s\right)=\frac{1}{2\pi }\frac{\partial }{\partial \text{s}}p_{H}\left(\vec{\xi },s\right),$$

where *h*_*H*_(*s*) = −1/2*π*∫_−*∞*_^*∞*^ *j* sgn⁡(*σ*)exp⁡(*j*2*πσs*)*dσ*. The second step in the inversion is the backprojection of filtered projections resulting from the application of Hilbert filter. Mathematically,

(4) $$f\left(\vec{x}\right)={\;\frac{1}{4\pi }\overset{2\pi }{\underset{0}{\int }}\frac{\partial }{\partial \text{s}}p_{H}\left(\vec{\xi },s\right)|}_{s=\vec{x}\cdot \vec{\xi }}d\theta .$$

### 2.2. Fan-Beam Projections

**Figure 2:**
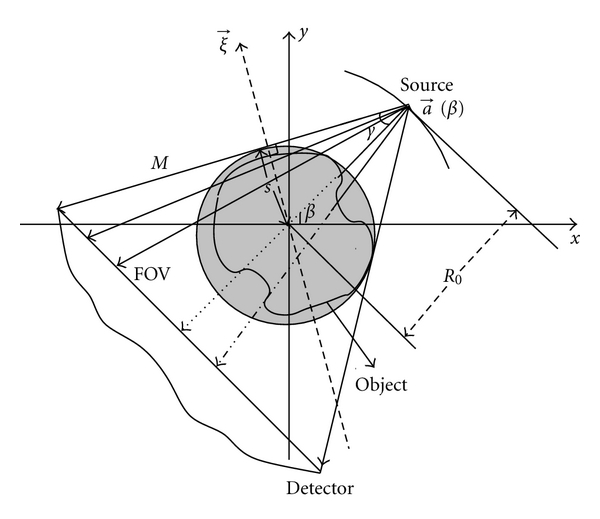
Relation between the fan-beam and parallel-beam geometry.

The fan-beam projections are obtained by using the fan-beam sampling by an equi-angular curved or equispaced collinear detector arrays. The following discussion makes no assumption about detector geometry. The fan-beam projection are measured by moving the X-ray source along a circular trajectory as shown in Figure 2. The source trajectory can be parameterized by an angular parameter *β* and is given by $\vec{a}(\beta )=(R_{0}\cos\beta ,\;R_{0}\sin\beta )$ where *R*_0_ is the radius of the circle. The fan-beam projections can be represented by

(5) $$\begin{array}{c} g\left(\beta ,\vec{\alpha }\right)=\overset{\infty }{\underset{0}{\int }}f\left(\vec{a}\left(\beta \right)+t\vec{\alpha }\right)dt,\;\;\vec{\alpha }\in S^{1} \end{array},$$

where **S** ^1^ is the set of all possible unit vectors in 2D space.

### 2.3. Relation between the Parallel Beam Projections and Fan-Beam Projections

Recalling that *p*_*H*_ denotes the Hilbert transform of the 2D Radon transform *p* of *f*. The line *M* corresponds to parallel beam ray and fan-beam ray as shown in Figure 2. Mathematical representation of this line satisfies *s*  =$\;\vec{a}(\beta )\cdot \vec{\xi }$, where $\vec{a}(\beta )$ is a point on this line. The value of *p*_*H*_ corresponding to this line can be obtained from $g_{H}(\beta ,\vec{\xi })$, and the relation between *p*_*H*_ and $g_{H}(\beta ,\vec{\xi })$ is given by

(6) $$\begin{array}{c} {p_{H}\left(\vec{\xi },s\right)|}_{s=\vec{a}(\beta )\cdot \vec{\xi }}=g_{H}\left(\beta ,\vec{\xi }\right), \end{array}$$

where

(7) $$\begin{array}{c} g_{H}\left(\beta ,\vec{\xi }\right)=-\overset{}{\underset{S^{1}}{\int }}h_{H}\left(\vec{\xi }\cdot \vec{\alpha }\right)g\left(\beta ,\vec{\alpha }\right)d\vec{\alpha }. \end{array}$$

**Figure 3:**
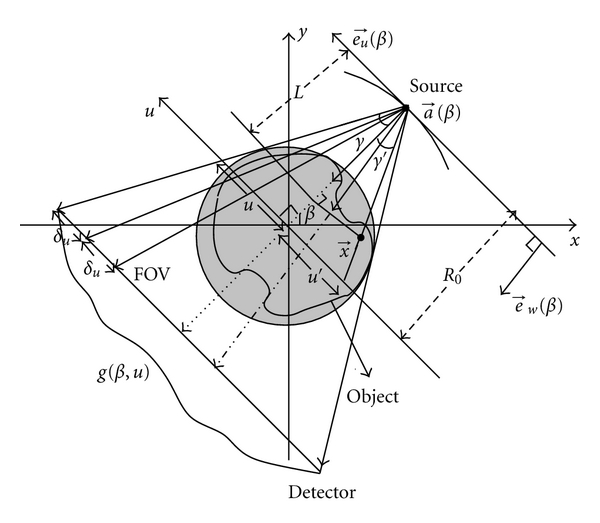
Fan-beam equispaced linear array detector scanning geometry.

This relation is given by Hamaker et al. [21]. The use of this relation has been explained through the parameterization of $\vec{\alpha }=\cos\gamma {\vec{e}}_{w}+\sin\gamma {\vec{e}}_{u}$ and $\vec{\xi }=-\sin\theta {\vec{e}}_{w}+\cos\theta {\vec{e}}_{u}$ [6], where ${\vec{e}}_{u}$ and ${\vec{e}}_{w}$ are unit vectors and these vectors are illustrated in Figure 3. The proof of (6) can be found in [6, 21]. Using (6), we derive two reconstruction algorithms with no backprojection weight for fan-beam equispace linear array detector. 

### 2.4. First Algorithm with No Backprojection Weight for Equispace Linear Array Detector

Let *g*(*β*, *u*) be the fan-beam projections for equispace detector and *p*(*θ*, *s*) be the parallel-beam projections. Using change of variable *γ* = tan^−1^(*u*/*R*_0_) in (7), we define Hilbert transform of *g*(*β*, *u*)

(8) $$\begin{array}{c} g_{H}\left(\beta ,u^{\prime }\right)=\frac{1}{\pi }\sqrt{R_{0}^{2}+u^{\prime 2}}\overset{u_{m}}{\underset{-u_{m}}{\int }}\frac{g\left(\beta ,u\right)}{u\prime -u}\frac{1}{\sqrt{R_{0}^{2}+u^{2}}}du. \end{array}$$

From the relation of Hamaker (6), *θ* = *β* − tan^−1^(*u*′/*R*_0_), and $s=R_{0}u\prime /\sqrt{R_{0}^{2}+u^{\prime 2}}$, we have *g*_*H*_(*β*, *u*′) = *p*_*H*_(*β* − tan^−1^(*u*′/*R*_0_), *R*_0_ *u*′/(*R*_0_^2^ + *u* ^′2^)). The derivative of *g*_*H*_(*β*, *u*′) with respect to *u*′ and *β* using chain rule is given by

(9) $$\begin{array}{c} \frac{\partial }{\partial u^{\prime }}g_{H}\left(\beta ,u^{\prime }\right)=\left[\frac{\partial s}{\partial u^{\prime }}\frac{\partial }{\partial s}+\frac{\partial \theta }{\partial u^{\prime }}\frac{\partial }{\partial \theta }\right]p_{H}\left(\theta ,s\right),\\ \frac{\partial }{\partial \beta }g_{H}\left(\beta ,u^{\prime }\right)=\left[\frac{\partial \theta }{\partial \beta }\frac{\partial }{\partial \theta }\right]p_{H}\left(\theta ,s\right). \end{array}$$

Since the parallel beam parameters are functions of the fan-beam parameters,  ∂*s*/∂*u*′, ∂*θ*/∂*u*′, and ∂*θ*/∂*β* are given by

(10) $$\begin{array}{c} \frac{\partial s}{\partial u\prime }=\frac{R_{0}^{3}}{R_{0}^{2}+u^{\prime 2}}\frac{1}{\sqrt{R_{0}^{2}+u^{\prime 2}}},\\ \;\frac{\partial \theta }{\partial u\prime }=\frac{-R_{0}}{R_{0}^{2}+u^{\prime 2}},\;\begin{array}{c} \frac{\partial \theta }{\partial \beta }=1. \end{array} \end{array}$$

Substituting (10) in (9), we get

(11) $$\begin{array}{cc} \frac{\partial }{\partial u^{\prime }}g_{H}\left(\beta ,u\prime \right) & =\left[\frac{R_{0}^{3}}{R_{0}^{2}+u^{\prime 2}}\frac{1}{\sqrt{R_{0}^{2}+u^{\prime 2}}}\frac{\partial }{\partial s}+\frac{-R_{0}}{R_{0}^{2}+u^{\prime 2}}\frac{\partial }{\partial \theta }\right]\\ & \;\times p_{H}\left(\beta -\tan^{-1}\frac{u\prime }{R_{0}},\frac{R_{0}u\prime }{R_{0}^{2}+u^{\prime 2}}\right), \end{array}$$

(12) $$\begin{array}{cc} \frac{\partial }{\partial \beta }g_{H}\left(\beta ,u^{\prime }\right) & =\frac{\partial }{\partial \theta }p_{H}\left(\beta -\tan^{-1}\frac{u^{\prime }}{R_{0}},\frac{R_{0}u^{\prime }}{R_{0}^{2}+u^{\prime 2}}\right). \end{array}$$

Substituting (12) in (11), (11) can be written as

(13) $$\begin{array}{cc} \frac{\partial }{\partial u^{\prime }}g_{H}\left(\beta ,u^{\prime }\right) & =\frac{R_{0}}{R_{0}^{2}+u^{\prime 2}}[\frac{\partial }{\partial s}p_{H}\left(\beta -\tan^{-1}\frac{u^{\prime }}{R_{0}},\frac{R_{0}u^{\prime }}{R_{0}^{2}+u^{\prime 2}}\right)\\ & \;\;\;\;\;\;\times \frac{R_{0}^{2}}{\sqrt{R_{0}^{2}+u^{\prime 2}}}-\frac{\partial g_{H}\left(\beta ,u\prime \right)}{\partial \beta }]. \end{array}$$

In (4), (∂/∂*s*)*p*_*H*_ is needed to reconstruct the function *f*, therefore (13) can be rewritten as

(14) $$\begin{array}{cc} & \frac{\partial }{\partial s}p_{H}\left(\beta -\tan^{-1}\frac{u^{\prime }}{R_{0}},\frac{R_{0}u^{\prime }}{R_{0}^{2}+u^{\prime 2}}\right)\\ & \;=\left(\frac{\partial }{\partial u^{\prime }}g_{H}\left(\beta ,u^{\prime }\right)\frac{R_{0}^{2}+u^{\prime 2}}{R_{0}}+\frac{\partial }{\partial \beta }g_{H}\left(\beta ,u^{\prime }\right)\right)\frac{\sqrt{R_{0}^{2}+u^{\prime 2}}}{R_{0}^{2}}. \end{array}$$

This result is used in (4) to get the first fan-beam inversion formula with no backprojection weight for equispace linear array detector. The inversion formula is given by

(15) $$\begin{array}{cc} & f\left(\vec{x}\right)=\frac{1}{4\pi }\overset{2\pi }{\underset{0}{\int }}[\left(\frac{\partial }{\partial u^{\prime }}g_{H}\left(\beta ,u^{\prime }\right)\frac{R_{0}^{2}+{u^{\prime }}^{2}}{R_{0}}+\frac{\partial }{\partial \beta }g_{H}\left(\beta ,u^{\prime }\right)\right)\\ & \;\;\;\;\;\;\times \frac{\sqrt{R_{0}^{2}+{u\prime }^{2}}}{R_{0}^{2}}]d\theta , \end{array}$$

where *β* = *θ* − tan^−1^(*u*′/*R*_0_) and $u\prime =\vec{x}\cdot \vec{\xi }R_{0}/\sqrt{R_{0}^{2}-{\left(\vec{x}\cdot \vec{\xi }\right)}^{2}}$. The integration in this formula being over *θ*, it is a parallel-beam backprojection. Equation (15) is applicable for full scan, short scan, and very short scan. For the short scan and over scan, (15) degenerates to the 180° parallel-beam reconstruction. In the case of super short scan, You and Zeng [8] suggested the redundancy weight similar to Noo's redundancy weighting. If the scanning range is [0  *π*], the redundancy weight can be calculated as follows:

(16) $$\begin{array}{c} w\left(\beta ,u^{\prime }\right)=wi\left(1-0.5wa\right) \end{array},$$

where *wi* is equal to 1 if *β* = (*θ* + tan^−1^(*u*′/*R*_0_)) − floor((*θ* + tan^−1^ (*u*′/*R*_0_))/2*π*)∗2*π* ≤ *π* otherwise *wi* = 0 and *wa* is equal to 1 if *β* = (*θ* − tan^−1^(*u*′/*R*_0_) + *π*) − floor((*θ* − tan^−1^(*u*′/*R*_0_) + *π*)/2*π*)∗2*π* ≤ *π* otherwise *wa* is equal to 0. The reconstruction formula for this scanning range is given by

(17) $$\begin{array}{cc} f\left(\vec{x}\right)= & \frac{1}{2\pi }\overset{\pi }{\underset{0}{\int }}w\left(\beta ,u^{\prime }\right)[(\frac{\partial }{\partial u^{\prime }}g_{H}\left(\beta ,u^{\prime }\right)\frac{R_{0}^{2}+u^{\prime 2}}{R_{0}}\;\\ & \;\;\;\;\;\;\;\;\;+\frac{\partial }{\partial \beta }g_{H}\left(\beta ,u^{\prime }\right))\frac{\sqrt{R_{0}^{2}+{u\prime }^{2}}}{R_{0}^{2}}]d\theta . \end{array}$$

### 2.5. Second Fan-Beam Algorithm with No Backprojection Weight for Equispace Linear Array Detector

Noo et al. [6] have proposed Hilbert FBP fan-beam inversion formula which can also reconstruct with less than short scan. The inversion formula is given by

(18) $$\begin{array}{c} f\left(\vec{x}\right)=\frac{1}{2\pi }\overset{2\pi }{\underset{0}{\int }}\frac{1}{L}{\left[w\left(\beta ,\vec{x}\right)g_{H}\left(\beta ,u^{\prime }\right)\right]}_{u^{\prime }=u^{\prime }(\beta ,\vec{x})}d\beta , \end{array}$$

where

(19) $$\begin{array}{c} \begin{array}{cc} g_{H}\left(\beta ,u^{\prime }\right) & =\overset{um}{\underset{-um}{\int }}h_{H}\left(u^{\prime }-u\right)\frac{R_{0}}{\sqrt{R_{0}^{2}+u^{2}}}\\ & \;\;\times \left(\frac{\partial }{\partial \beta }+\frac{R_{0}^{2}+u^{2}}{R_{0}}\frac{\partial }{\partial u}\right)g\left(\beta ,u\right)du, \end{array} \end{array}$$

(20) $$\begin{array}{c} u^{\prime }\left(x,y,\beta \right)=\frac{R_{0}\vec{x}\cdot {\vec{e}}_{u}}{R_{0}+\vec{x}\cdot {\vec{e}}_{w}},\\ L=R_{0}+\vec{x}\cdot {\vec{e}}_{w}. \end{array}$$

The ray defined by $\left(\beta ,u\prime (\vec{x},\beta )\right)$ contains the point $\vec{x}$. *L* is the distance from source position obtained by projecting the point $\vec{x}$ on the ray passing through the origin. Illustration of *u*′ and *L* are shown Figure 3. Since the redundancy weighting *w*(*β*, *u*′) in (18) applies after the Hilbert filtering, it can be applied in the image domain as part of the backprojection step. This is not feasible with the standard FBP formula because redundancy weight is applied before filtering.

**Figure 4:**
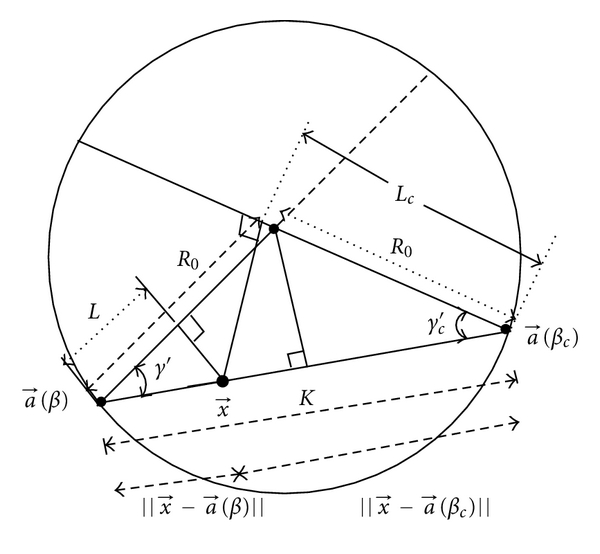
Illustration of a fan-beam scanning geometry showing redundantly measured line integral along line *K*. The line integral along *K* corresponds to source positions *a*(*β*) and *a*(*β*_*c*_).

Dennerlein et al. [7] have suggested the valid redundancy weight for full scan,

(21) $$\begin{array}{c} w\left(\beta ,\vec{x}\right)=\frac{||\vec{x}-\vec{a}\left(\beta \right)||}{2R_{0}\cos\;\gamma \prime } \end{array},$$

where *γ*′ = arctan(*u*′/*R*_0_). From the geometry shown in Figures 3 and 4, it can be proved that *w*(*β*, *γ*′) + *w*(*β*_*c*_, *γ*_*c*_′) = 1, where *w*(*β*, *γ*′) and *w*(*β*_*c*_, *γ*_*c*_′) are the redundancy weights corresponding to the redundant line *K* which passes through the point $\vec{x}$. Equation (21), in terms of equispace array geometry parameters, can be written as

(22) $$\begin{array}{c} w\left(\beta ,\vec{x}\right)=L\frac{R_{0}^{2}+u^{\prime 2}}{2R_{0}^{3}}, \end{array}$$

where $L=||\vec{x}-\vec{a}(\beta )||\cos\gamma \prime =R_{0}+\vec{x}\cdot {\vec{e}}_{w}$. The second fan-beam inversion formula with no backprojection weight for equispace linear array detector is obtained by substituting (22) in (18) and is given as

(23) $$\begin{array}{c} f\left(\vec{x}\right)=\frac{1}{2\pi }\overset{2\pi }{\underset{0}{\int }}\frac{R_{0}^{2}+u^{\prime 2}}{2R_{0}^{3}}g_{H}{\left(\beta ,u^{\prime }\right)}_{u^{\prime }=u^{\prime }(\beta ,\vec{x})}d\beta , \end{array}$$

where *g*_*H*_ is given by (19) and $u\prime =R_{0}\vec{x}\cdot {\vec{e}}_{u}/{(R}_{0}+\vec{x}\cdot {\vec{e}}_{w})$.

Thus, the backprojection weight (*L*) in (18) can be eliminated for each ray that corresponds to a line integral that is measured twice by weighting each ray with function $w(\beta ,\vec{x})$ of (21) during the backprojection for short scan or super short scan. However, it does not allow elimination of the weight for singly measured rays (i.e, in case of short scan or a super short scan).

## 3. New FDK-Type Formulae

Feldkamp et al. [13] and Wang et al. [14] have developed 3D CB CT reconstruction algorithms for circular and helical trajectories, respectively, from the conventional fan-beam reconstruction formula. Similarly, in this section, we propose two new FDK-type inversion formulae for 3D CB reconstruction with the flat panel array detector CB geometry which are obtained by heuristic extension of the fan-beam inversion formulae with no backprojection weight. These formulae are the counterpart of the formulae for cylindrical array detector given by Narasimhadhan et al. [22, 23] for circular and helical trajectory CB CT reconstructions.

### 3.1. Cone-Beam Geometry

Let *f*(*x*, *y*, *z*) be the object density to be reconstructed and *g*(*β*, *u*, *v*) be the flat panel detector CB projection data collected with a helical scan trajectory given by $\vec{a}(\beta )={\left[R_{0}\cos\beta ,\;R_{0}\sin\beta ,h\beta \right]}^{T}$ where *R*_0_ is the radius of the source trajectory, *h* = *P*/2*π*, and *P* represents the helical pitch. To describe the CB data for a flat detector, we introduce the local detector coordinates (*u*, *w*, *v*) with unit vectors: ${\vec{e}}_{u}(\beta )={\left[-\sin\beta ,\;\cos\beta ,0\right]}^{T}$, ${\vec{e}}_{w}(\beta )={\left[-\cos\beta ,\;-\sin\beta ,0\right]}^{T}$, and ${\vec{e}}_{v}(\beta )={\left[0,0,1\right]}^{T}$. Here ${\vec{e}}_{w}(\beta )$ points from the source point $\vec{a}(\beta )$ to the center of the detector and the unit vectors ${\vec{e}}_{u}(\beta )$ and ${\vec{e}}_{v}(\beta )$ span the detector. The CB projection for flat panel detector geometry is given by

(24) $$\begin{array}{cc} & g\left(\beta ,u,v\right)\\ & \;=\overset{\infty }{\underset{0}{\int }}f\left(\vec{a}\left(\beta \right)+t\left(\frac{u{\vec{e}}_{u}\left(\beta \right)+D{\vec{e}}_{w}\left(\beta \right)+v{\vec{e}}_{v}\left(\beta \right)}{\sqrt{u^{2}+D^{2}+v^{2}}}\right)\right)dt, \end{array}$$

where *D* is the source to detector distance.

### 3.2. New FDK Formulae for Helical and Circular CB Geometry

To simplify the formulae given below, we assume that the flat panel virtual detector is parallel to the actual detector panel at a distance *R*_0_ from $\vec{a}(\beta )$ and hence contains the *z*-axis. Scaling all equations by substituting *u* → *R*_0_ *u*/*D* and *v* → *R*_0_ *v*/*D* is sufficient to describe the case of a flat panel array detector located at a distance *D* from the vertex.

#### 3.2.1. First FDK Formula with No Backprojection Weight for Helical Trajectory (*HFDKW*-1)

Let *h*_*H*_(·) be Hilbert filter kernel in spatial domain. The HFDKW-1 formula is obtained from (15) by the standard FDK extension technique for a particular slice *z*,

(25) $$\begin{array}{cc} & \hat{f}\left(x,y,z\right)\\ & \;=\frac{1}{4\pi }\overset{(z/h)+\pi }{\underset{(z/h)-\pi }{\int }}\frac{R_{0}^{2}+{u^{\prime }}^{2}}{R_{0}^{3}}\left(\overset{\infty }{\underset{-\infty }{\int }}\hat{g}\left(\beta ,u,v^{\prime }\right)h_{H}\left(u^{\prime }-u\right)du\right)d\theta , \end{array}$$

where

(26) $$\begin{array}{cc} & \hat{g}\left(\beta ,u,v\right)\\ & \;\begin{array}{c} =\frac{R_{0}}{\sqrt{R_{0}^{2}+u^{2}+v^{2}}}\left(\frac{\partial }{\partial \beta }+\frac{R_{0}^{2}+u^{2}}{R_{0}}\frac{\partial }{\partial u}+\frac{uv}{R_{0}}\frac{\partial }{\partial v}\right)g\left(\beta ,u,v\right), \end{array} \end{array}$$

*β*(*x*, *y*, *θ*) = *θ* + *γ*′(*x*, *y*, *θ*), and *γ*′(*x*, *y*, *θ*) = arcsin ((−*x*sin⁡*θ* + *y*cos⁡*θ*)/*R*_0_). The variables *u*′ and *v*′ are computed by the following equations:

(27) $$\begin{array}{cc} u^{\prime }\left(x,y,\theta \right) & =\frac{R_{0}\left(-x\sin\beta \left(x,y,\theta \right)+y\cos\beta \left(x,y,\theta \right)\right)}{R_{0}-x\cos\beta -y\sin\beta \left(x,y,\theta \right)},\\ v^{\prime }\left(x,y,\theta \right) & =\frac{R_{0}\left(z-h\beta \left(x,y,\theta \right)\right)}{R_{0}-x\cos\beta \left(x,y,\theta \right)-y\sin\beta \left(x,y,\theta \right)}. \end{array}$$

#### 3.2.2. Second FDK Formula with No Backprojection Weight for Helical Trajectory (*HFDKW*-2)

The HFDKW-2 formula is obtained from the (23) by standard FDK extension technique

(28) $$\begin{array}{cc} & \hat{f}\left(x,y,z\right)\\ & \begin{array}{c} \;=\frac{1}{4\pi }\overset{(z/h)+\pi }{\underset{(z/h)-\pi }{\int }}\frac{R_{0}^{2}+u^{\prime 2}}{R_{0}^{3}}\overset{\infty }{\underset{-\infty }{\int }}\hat{g}\left(\beta ,u,v^{\prime }\right)h_{H}\left(u^{\prime }-u\right)du\;d\beta , \end{array} \end{array}$$

where

(29) $$\begin{array}{cc} u^{\prime }\left(x,y,\beta \right) & =\frac{R_{0}\left(-x\sin\beta +y\cos\beta \right)}{R_{0}-x\cos\beta -y\sin\beta },\\ v^{\prime }\left(x,y,z,\beta \right) & =\frac{R_{0}\left(z-h\beta \right)}{R_{0}-x\cos\beta -y\sin\beta }, \end{array}$$

and $\hat{g}(\beta ,u,v)$ is given by (26).

Note that the integration in second formula in (28) is over *β* and therefore it is a fan-beam backprojection while the integration in first HFDKW formula (25) being over *θ* results in a parallel-beam backprojection. The first HFDKW formula (25) can be modified to include redundancy weight for partial scan data as given by You and Zeng [8] (25).

The conventional FDK formula involves a weighted backprojection. The weight *L* ^−2^ in the backprojection, where *L* is the distance from source position obtained by projecting the point $\vec{x}$ on the ray passing through the origin, is therefore position-dependent. The weight (*R*_0_^2^ + *u* ^2^)/*R*_0_^3^ required in the HFDKW-2 can be evaluated and multiplied with filtered projection data before the backprojection. Since there is no position dependent weight in the backprojection step, HFDKW-2 is computationally more efficient than the conventional FDK algorithm. HFDKW-1 algorithm performs an additional interpolation in the backprojection, hence the computational complexity of the HFDKW-1 is more than the conventional HFDK and HFDKW-2 algorithms.

#### 3.2.3. FDK Formulae with No Backprojection Weight for Circular Trajectory

The HFDKW-1 and HFDKW-2 algorithms can be reduced to circular FDK algorithms with no backprojection weight by setting the helical pitch *P* = 0. The resulting two algorithms are named as CFDKW-1 and CFDKW-2, respectively. It is seen from the Figure 7 that the CFDKW algorithms, Hu's algorithm, and T-FDK algorithm give similar performance in terms of axial intensity drop. This motivates us to develop an efficient approach using the estimate of missing term as given by Zhu et al. [20].

## 4. Efficient Approaches to Reduce the Axial Intensity Drop in Circular CB CT

It has been shown by reformulating Grangeat's algorithm for circular CB trajectory that the reconstruction of the true object *f* can be written as the sum of three terms [19]:

(30) $$\begin{array}{c} \hat{f}={\hat{f}}_{\text{FDK}}+{\hat{f}}_{H}+{\hat{f}}_{N} \end{array},$$

where ${\hat{f}}_{\text{FDK}}$ is the reconstructed object using FDK algorithm, ${\hat{f}}_{H}$ is Hu's correction term, and ${\hat{f}}_{N}$ is the missing term. Hu's term represents the information contained in a circular CB scan but not utilized in the FDK reconstruction. First two terms of the equation are obtained using the circular CB data [19]. The third term can be obtained in different ways. One approach is to estimate the missing term from the available circular CB scan data as proposed by Zhu et al. [20], which includes all the three terms in (30).

In this section, we propose to use the circular FDK inversion with no backprojection weight (CFDKW algorithms) and the estimate of missing term ${\hat{f}}_{N}$ using Zhu et al.'s to improve the axial intensity profile of the reconstructed image. Thus, the reconstructed object $\hat{f}$ of the true object *f*, with the two algorithms, CFDKW-1 and CFDKW-2, respectively, can be written as:

(31) $$\begin{array}{cc} \hat{f} & ={\hat{f}}_{\text{CFDKW-}1}+{\hat{f}}_{N},\\ \hat{f} & ={\hat{f}}_{\text{CFDKW-}2}+{\hat{f}}_{N}. \end{array}$$

These two efficient algorithms are called EM-1 and EM-2, respectively, for further reference. The estimation of missing term is based on Hu's theory [19] and Grangeat's theory [24]. The formula for estimating the missing term [20] is given by

(32) $${\hat{f}}_{N}\left(\vec{x}\right)=-\frac{1}{4\pi^{2}}\frac{R_{0}^{2}+z^{2}}{R_{0}^{2}}\left(1-\frac{\sqrt{R_{0}^{2}-z^{2}}}{R_{0}}\right)\overset{2\pi }{\underset{0}{\int }}\hat{g}\left(\beta ,z\right)d\beta ,$$

where $\hat{g}\left(\beta ,z\right)=(\partial /\partial z^{2})\int_{-\infty }^{\infty }(R_{0}/\sqrt{R_{0}^{2}+u^{2}+z^{2})}g(\beta ,u,z)du$.

We show through simulations that the efficient algorithms and Zhu et al.'s [20] algorithm give qualitatively same intensity profile and improvement in intensity drop. 

## 5. Simulation Results

**Figure 5:**
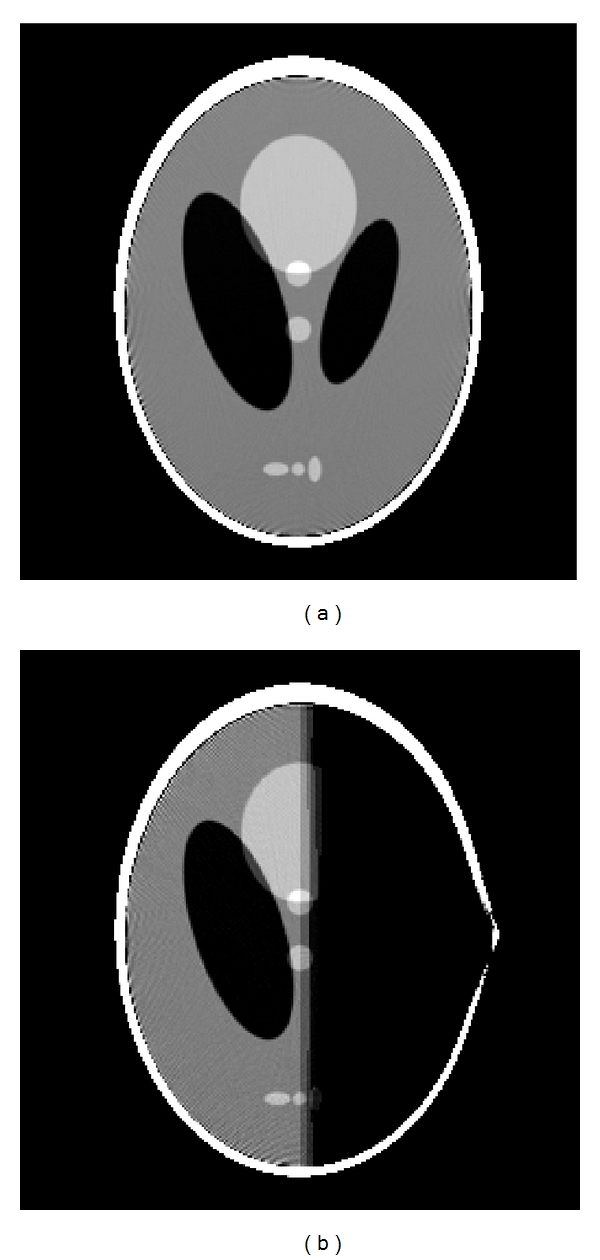
Reconstructed slices at *z* = −0.25 of Shepp-Logan low-contrast phantom using HFDKW-1 formula for (a) short scan segment on a helical trajectory. (b) Less than short scan segment. The value of pitch used is 0.125 m. Display window is [1 1.04].

Cone-beam circular and helical X-ray source trajectory scanner is used in the computer simulations. The radius of the circular and helical trajectory of the source *R*_0_ is 2.4 m and the object radius is 1 m for the simulations. Circular CB projection data is simulated with 450 uniformly spaced source positions over 2*π* interval, using 283 detector elements, and 283 detector rows. Helical CB projection data is simulated with 450 uniformly spaced source positions over 2*π* interval, using 283 detector elements, and 29 (111) detector rows with pitch value *P* = 0.125 m (0.5 m). Image matrix for a slice of the 3D phantom is 256 × 256. Simulations are carried out on full, short, and very short scan data obtained from 3D Shepp-Logan low contrast phantom [14] for HFDKW algorithms. HFDKW-2 algorithm is based on fan-beam backprojection over 2*π* scan and therefore it can only handle full-scan reconstruction. HFDKW-1 is a more general algorithm which can accommodate both short scan as well as very short scan data.  Figure 5(a) shows the reconstructed slice at *z* = −0.25 of Shepp-Logan low-contrast phantom using HFDKW-1 for short scan (scan angle of *π* + fan angle) and Figure 5(b) shows the reconstruction with very short scan case (scan angle = *π*).

The 3D Shepp-Logan low-contrast phantom, ForBild head phantom and Defrise disk phantom, are used to illustrate the performance of the proposed algorithms for 3D reconstruction with circular scan trajectories under different conditions. Defrise disk phantom consists of nine ellipsoidal discs stacked along the *z* direction. Each disc has a uniform attenuation coefficient of 0.7, and the ellipsoid has a diameter of 1.2 m and a thickness of 0.12 m, with a distance of 0.2 m between discs. We evaluate the performance of the CFDKW algorithms in terms of longitudinal or axial intensity drop. We compare the CFDKW algorithms with three different algorithms: the FDK algorithm which is only the first term of (30), Hu's algorithm (consists of first two terms of (30)) [19], and T-FDK algorithm which has been developed heuristically with a structure of shift-invariant filtering [25]. We also illustrate the performance of the EM-1 and EM-2 in terms of reduction in the axial intensity drop, noise level, and computational complexity. We compare the EM-1 and EM-2 with Zhu et al.'s [20] algorithm (i.e, all the three terms in (30)).

### 5.1. Axial Intensity Drop

**Figure 6:**
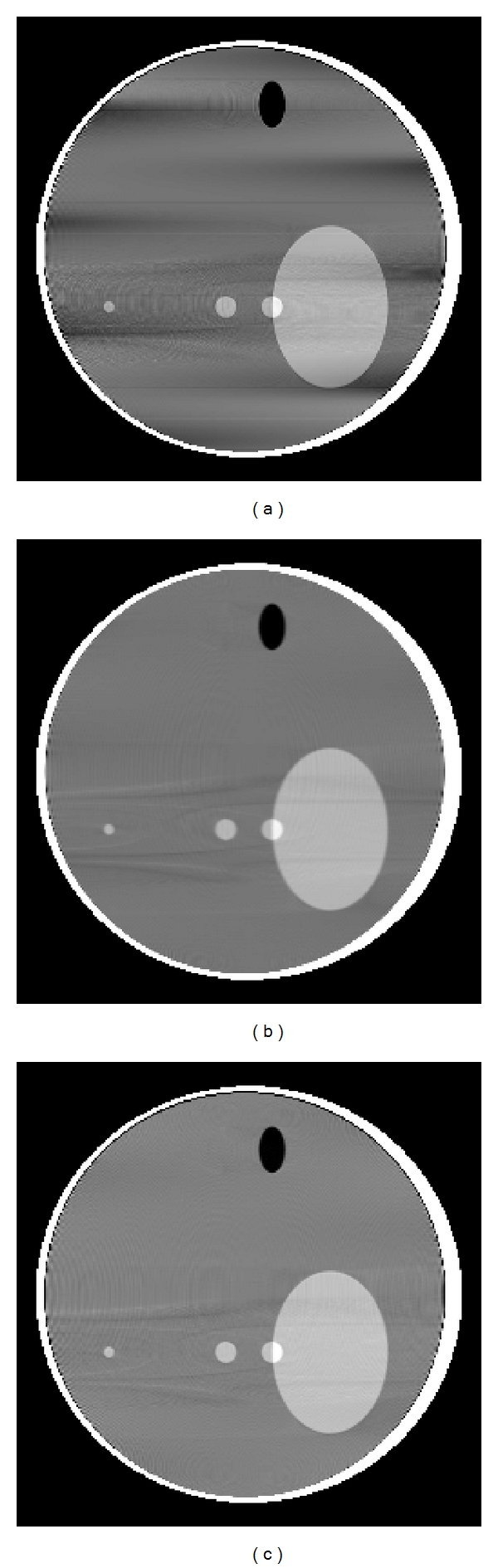
Slices of Shepp-Logan low-contrast phantom at *x* = 0. Illustration of axial intensity drop due to large value of pitch 0.5 m in full-scan reconstruction (a) standard helical FDK. (b) HFDKW-1 algorithm and (c) HFDKW-2 algorithm. Display window is [1 1.04].

The axial intensity drop and variation become significant in conventional helical FDK algorithms due to the approximate nature of the algorithm when large helical pitch is used. We reconstructed the slices of 3D Shepp-Logan low-contrast phantom with helical pitch 0.5 m using FDK, HFDKW-1, and HFDKW-2 algorithms. It is observed from Figures 6(a), 6(b), and 6(c) that the reconstructed image using the helical FDK shows the axial intensity drop and variation compared to the reconstructed images using HFDKW algorithms. Thus, HFDKW algorithms can be applicable for large value of pitch.

**Figure 7:**
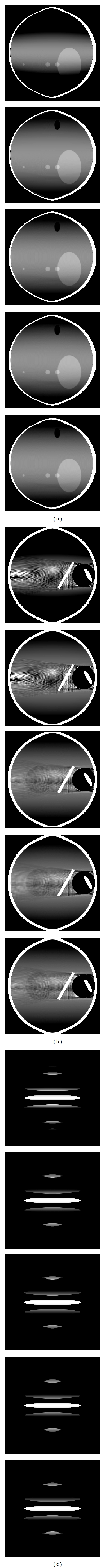
From left to right column, reconstructed slices of Shepp-Logan low contrast, ForBild head and Defrise disk phantom, respectively, at *x* = 0. From top row to bottom row, reconstruction with FDK algorithm, Hu's algorithm, T-FDK algorithm, CFDKW-1, and CFDKW-2 respectively. Display windows are [0.98 1.05], [1.01 1.09] and [0.5 0.7] for Shepp-Logan, ForBild head and Defrise disk phantom, respectively.

**Figure 8:**
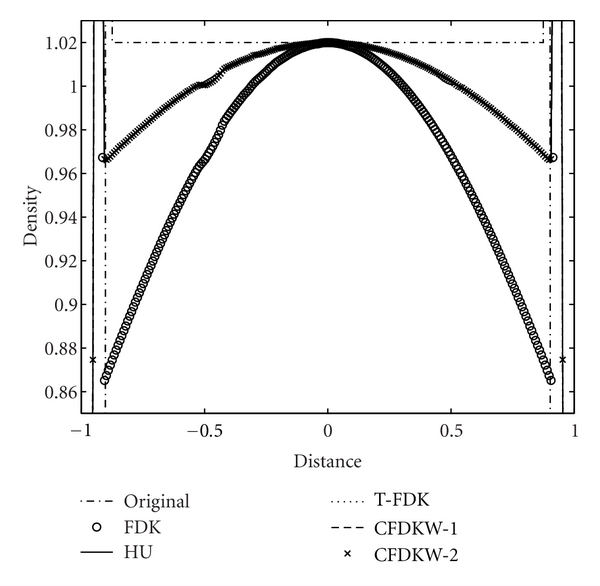
Illustration of axial intensity drop in full-scan reconstructions of Shepp-Logan low-contrast phantom, vertical intensity profile of the Shepp-Logan low-contrast phantom at *z* = 0 in Figure 7.

**Figure 9:**
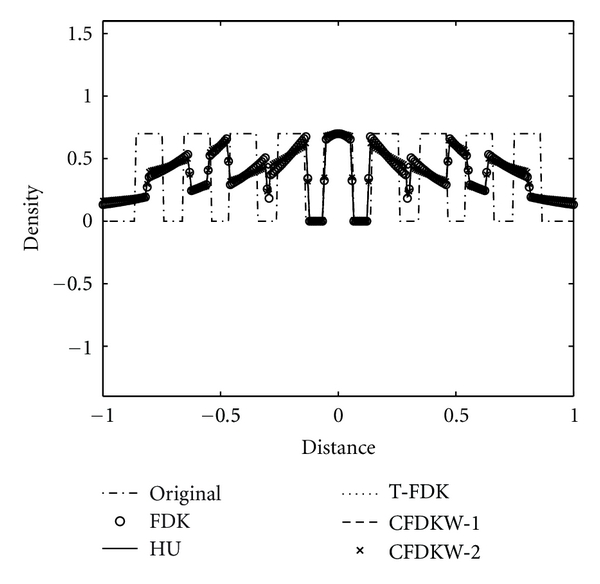
Illustration of axial intensity drop in full-scan reconstructions of Defrise disk phantom, vertical intensity profile of the Defrise disk phantom at *z* = 0 in Figure 7.

We study the axial intensity drop associated with the circular FDK algorithms due to data incompleteness of the circular trajectory in 3D reconstruction. The Shepp-Logan low-contrast phantom, ForBild phantom, and the Defrise disk phantom are used to illustrate the performance of the CFDKW algorithms, in comparison with FDK, Hu's, and T-FDK algorithms. The first column of the Figure 7 shows the reconstruction of the Shepp-Logan low-contrast phantom with full scan circular trajectory using FDK, Hu's, T-FDK and CFDKW algorithms. It is observed that the drop in density in the case of Hu's, T-FDK, CFDKW-1, and CFDKW-2 in Figure 7 is same and significantly less than the circular FDK. This can be clearly seen in the intensity profiles in the Figure 8. The second column of the Figure 7 shows the reconstruction of ForBild phantom which has high-density object in the central region leading to artifacts. It is seen in Figure 7 that the reconstructed images of ForBild head phantom have white and black streaks using the FDK and Hu's algorithms. These streaks are significantly reduced in the reconstructed images using the CFDKW and T-FDK algorithms. The Defrise disk phantom is used to show the performance of the algorithms to reconstruct sharp change in the axial intensity. The strong high-frequency components in the axial direction due to sharp change in density represents the most challenging aspect of the reconstruction using circular CB data.  Figure 9 shows the 1D axial intensity profile of Defrise disk phantom illustrating the observations made earlier in Figure 7. It is seen from the axial intensity profile in Figure 9 that only the central disk is reconstructed properly by all the algorithms.

**Figure 10:**
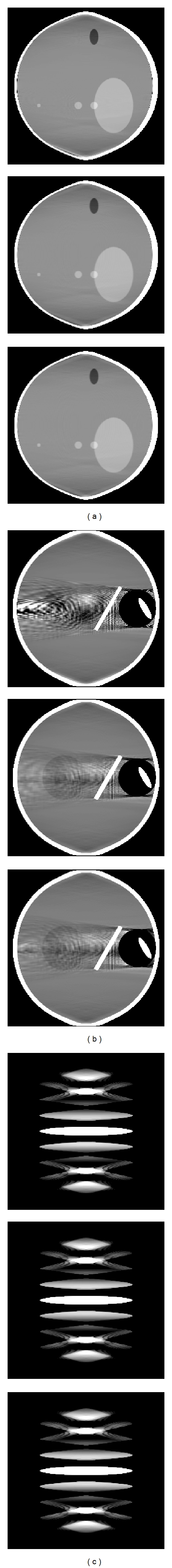
From left to right column, reconstructed slices of the Shepp-Logan low contrast, ForBild head and Defrise disk phantom, respectively, at *x* = 0. From top row to bottom row, reconstruction with Zhu et al.'s algorithm, EM-1 (CFDKW-1 + FN), and EM-2 (CFDKW-2 + FN), respectively. Display windows are [0.98 1.05], [1.01 1.09] and [0.5 0.7] for Shepp-Logan, ForBild head, and Defrise disk phantom, respectively.

**Figure 11:**
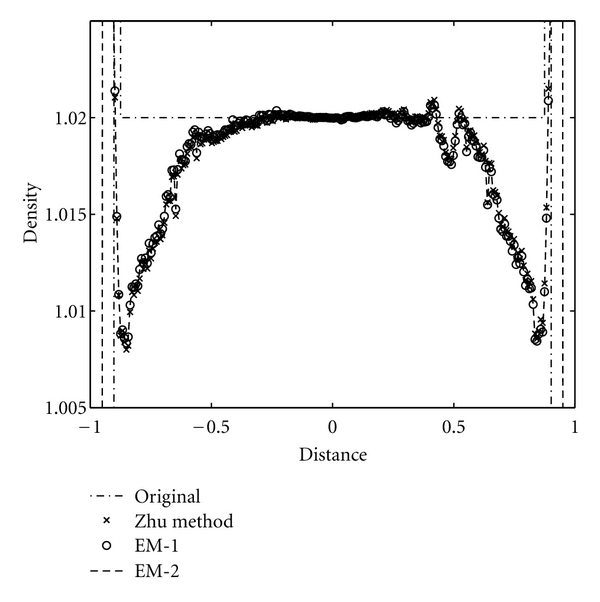
Illustration of axial intensity drop in full-scan reconstructions of Shepp-Logan low contrast phantom, vertical profile of the Shepp-Logan low contrast phantom at *z* = 0 in Figure 10.

**Figure 12:**
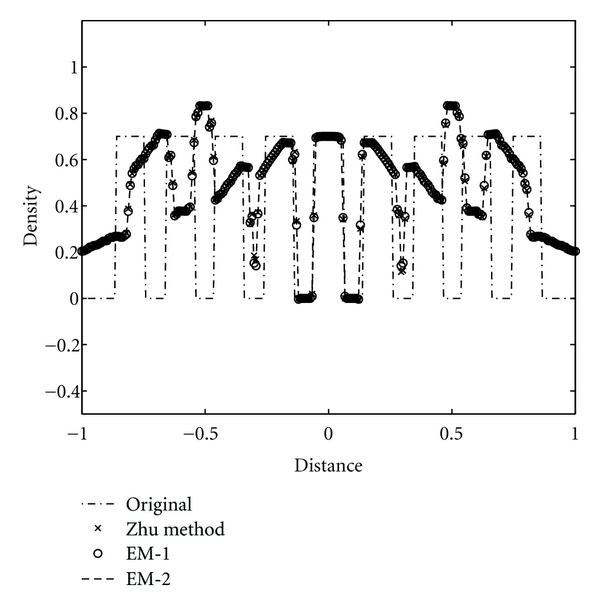
Illustration of axial intensity drop in full-scan reconstructions of Defrise disk phantom, vertical profile of the Defrise disk phantom at *z* = 0 in Figure 10.

We reconstruct the three phantoms using the EM algorithms and compare them with the reconstructions obtained by Zhu et al.'s algorithm.  Figure 10 shows the reconstruction using Zhu et al.'s algorithm, EM-1, and EM-2 algorithms of Shepp-Logan phantom in the first column, ForBild phantom in the second column and Defrise disk in the third column, respectively. It is seen qualitatively from the Figure 10 that Zhu et al.'s, EM-1, and EM-2 reconstruction algorithms give the same performance in terms of the axial intensity drop and significant improvement over reconstructions shown in Figure 7. This is also evident quantitatively from the axial intensity profiles shown in Figures 11 and 12 of the Shepp-Logan and Defrise disk phantoms in Figure 10, respectively.

### 5.2. Noise Performance

**Figure 13:**
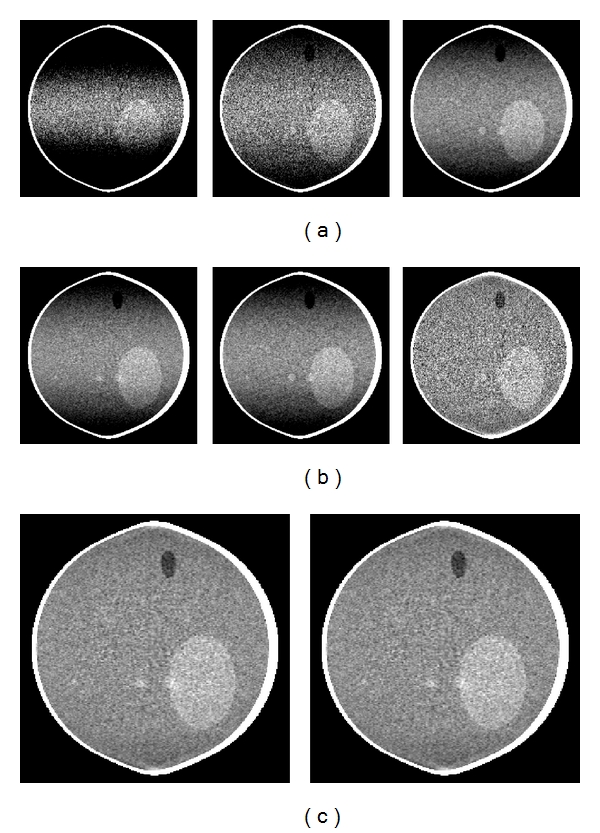
Slices of Shepp-Logan low contrast phantom at *x* = 0, reconstruction with (a) FDK algorithm, Hu's algorithm, T-FDK algorithm, (b) CFDKW-1, CFDKW-2, Zhu et al.'s algorithm, (c) EM-1 and EM-2 with added noise. Display window is [0.98 1.05].

We simulate noisy projection data using the Shepp-Logan low-contrast phantom with Poisson noise based on an emission of *N*_0_ = 3 × 10^5^ photons. The number of photons detected *N* was calculated by −ln⁡(*N*/*N*_0_). With this as the mean to a Poisson random process, *N* for the case of noisy projection is computed, corresponding noisy projection is then given by −ln⁡(*N*/*N*_0_). The image is reconstructed using the eight different algorithms to make a qualitative and quantitative evaluation of the CFDKW and the EM algorithms in comparison to other FDK algorithms as given in Table 1.  Figure 13 shows the reconstructed noisy images for the eight algorithms in the same order as given in Table 1. To compute the error variances of the images, the error images are obtained by taking the difference of reconstructed noisy images (Figure 13) and corresponding noise-free images as shown in Figures 7 and 10. The variances of error images are measured and the variances are given in the Table 1. It is seen from Table 1 that the noise level is reduced in the CFDKW and EM algorithms. This is due to the additional numerical differentiation step, which is usually implemented as two- or three-point difference, and these algorithms are based on Hilbert filtering which avoids the approximation introduced by the nonuniform cutoff frequency required in the ramp filter-based FBP algorithm. Since CFDKW-1 and EM-1 algorithms perform additional interpolation in the source direction in the backprojection, these algorithms give better noise performance among all algorithms.

### 5.3. Computational Complexity

We compute the execution time for FDK algorithm, Hu's term, missing term, Hu's algorithm, Zhu et al.'s algorithm, CFDKW-2, and EM-2 algorithms in MATLAB 7.0 on a Intel P4 desktop computer with a dual core 2.4 GHz processor platform. The algorithm modules considered for estimating the execution time are (1) 1D filtering of the CB projections and (2) backprojection of a single slice. The execution time of the reconstruction for FDK, Hu's term, missing term, Zhu et al.'s algorithm, CFDK-2, and EM-2 algorithms are computed using these modules. The execution times are given in the Table 2. CFDKW-2 does not have position-dependent weight in the backprojection, hence CFDKW-2 algorithm has less computational complexity than FDK and Hu's algorithms. The computational complexity of EM-2 algorithm is less than Zhu et al.'s algorithm. The EM-2 algorithm consists of the reconstruction of the image using CFDKW-2 algorithm and estimating the missing term, whereas Zhu et al.'s algorithm consists of the reconstruction of the image using conventional FDK, estimation of Hu's term, and estimation of missing term. Hence the computational complexity of EM-2 is less than Zhu et al.'s algorithm.

## 6. Conclusion

We have given two fan-beam algorithms with no backprojection weight for equispace linear array detector, which are counterparts of the algorithms with no backprojection weight for equi-angular detector geometry. Two new variants of FDK algorithms for circular and helical CB scan geometry with planar detector geometry are proposed. The CFDKW-1 and HFDKW-1 are applicable to full, short and very short scan data. The CFDKW-2 and HFDKW-2 deal only with the full scan data. The CFDKW and HFDKW algorithms are based on Hilbert filtering and have no position-dependent weights in the backprojection step and hence reduction in the axial intensity drop and variation when compared with the classical FDK algorithm. The CFDKW, T-FDK, and Hu's algorithms give identical performance in the reduction of axial intensity drop in the circular CB CT. CFDKW-2 algorithm is more efficient than the FDK and Hu's algorithm in terms of computational complexity. We have given efficient algorithms to reduce the axial intensity drop in the circular scan CB CT. These efficient algorithms and Zhu et al.'s algorithm give a similar performance in terms of the axial intensity drop. The EM-2 algorithm outperforms the other algorithms in terms of computational complexity. Noise performance is better in case of CFDKW and EM algorithms than conventional FDK, Hu's algorithm, T-FDK and Zhu et al.'s algorithms.

## Figures and Tables

**Table 1:** Variance of the noisy images in Figure 13 based on the noise-free images in Figure 7.

Algorithm	value × 10^−4^
FDK	1.3986
Hu's	1.3999
T-FDK	0.27443
CFDKW-1	0.24365
CFDKW-2	0.26443
Zhu et al.'s	1.3999
EM-1	0.24365
EM-2	0.26443

**Table 2:** Execution time (in seconds).

	Single slice	Total volume (256 × 256 × 256)		
	Backprojection	Filtering	Backprojection	Total

FDK	11.8	76.4	3020	3096.4
Hu's term	5.6	2.3	1433.6	1435.9
Missing term	5.3	2.4	1356.8	1359.2
Hu's algorithm	17.4	78.7	4453.6	4542.3
Zhu et al.'s algorithm	22.7	81.1	5811.2	5892.3
CFDKW-2	11	90	2816	2906
EM-2	16.3	92.4	4172.8	4265.2
